# Personal protective equipment for surgeons during COVID-19 pandemic: systematic review of availability, usage and rationing

**DOI:** 10.1002/bjs.11750

**Published:** 2020-08-24

**Authors:** Z M Jessop, T D Dobbs, S R Ali, E Combellack, R Clancy, N Ibrahim, T H Jovic, A J Kaur, A Nijran, T B O'Neill, I S Whitaker

**Affiliations:** Reconstructive Surgery and Regenerative Medicine Research Group, Swansea University Medical School, Institute of Life Science, University of Swansea, Swansea, UK; Welsh Centre for Burns and Plastic Surgery, Morriston Hospital, Swansea, UK

## Abstract

**Background:**

Surgeons need guidance regarding appropriate personal protective equipment (PPE) during the COVID-19 pandemic based on scientific evidence rather than availability. The aim of this article is to inform surgeons of appropriate PPE requirements, and to discuss usage, availability, rationing and future solutions.

**Methods:**

A systematic review was undertaken in accordance with PRISMA guidelines using MEDLINE, Embase and WHO COVID-19 databases. Newspaper and internet article sources were identified using Nexis. The search was complemented by bibliographic secondary linkage. The findings were analysed alongside guidelines from the WHO, Public Health England, the Royal College of Surgeons and specialty associations.

**Results:**

Of a total 1329 articles identified, 95 studies met the inclusion criteria. Recommendations made by the WHO regarding the use of PPE in the COVID-19 pandemic have evolved alongside emerging evidence. Medical resources including PPE have been rapidly overwhelmed. There has been a global effort to overcome this by combining the most effective use of existing PPE with innovative strategies to produce more. Practical advice on all aspects of PPE is detailed in this systematic review.

**Conclusion:**

Although there is a need to balance limited supplies with staff and patient safety, this should not leave surgeons treating patients with inadequate PPE.

## Graphical Abstract

This systematic review provides a comprehensive overview of all the issues surrounding personal protective equipment for surgeons during the COVID-19 pandemic. To overcome this global challenge needs an evidence-based, collaborative, innovative multidisciplinary approach.

**Graphical Abstract:**
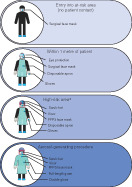

Better to be safe than sorry

## Introduction

In December 2019, clusters of patients presenting with severe pneumonia of unknown origin were reported in the city of Wuhan, Hubei Provence, China. Epidemiologically, these were linked to a seafood market in the city, and on 7 January 2020 the causative organism was identified, a novel coronavirus, now termed SARS-CoV-2^1^. In March 2020, the WHO declared a global pandemic^2^, which has gathered speed across the world despite increasingly more drastic non-pharmacological interventions (NPIs) to limit its spread. With one-quarter of the world's population now living under some form of government-mandated lockdown, and over three million documented cases worldwide, NPIs are the main public health measure that policymakers are using to reduce viral transmission^3,4^. Social distancing aims to flatten the curve of new infections, thereby avoiding a surge in demand on healthcare systems, but these effects may take weeks or months to manifest. Epidemiological modelling has shown that the pandemic could last for 12–18 months, and that social distancing may need to continue until a vaccine has been developed^3^.

The impact of this crisis on surgical services will be wide ranging^5^. Many thousands of patients worldwide have been deprived of surgical access, and are waiting to undergo elective and emergency surgical procedures. They will become part of the second and subsequent waves, with undoubted morbidity and mortality as a collateral effect of the COVID-19 pandemic^6,7^. Currently, the majority of evidence is of low quality, including case series and observational studies, with heterogeneous populations and surgical intervention groups. Fortunately, global collaborative initiatives have been launched, the COVIDSurg Collaborative^8^ being an excellent example, which aims to give guidance on how to deliver surgical services safely and effectively during the COVID-19 pandemic.

WHO modelling of personal protective equipment (PPE) for healthcare professionals has estimated that 89 million medical masks, 76 million gloves, 1·6 million goggles and 30 million gowns are required for the COVID-19 response each month^9^. China is the major producer and supplier of PPE globally, and its exports have come to a halt as the infection spreads domestically^10^. A combination of disruption to worldwide supply chains and international travel restrictions, combined with exceptionally high levels of demand, slow release of pandemic stocks as well as confusing and ever changing PPE guidelines, has led to a lack of PPE for frontline medical staff^11^, and confusion around when it is needed^12^. This systematic review summarizes the SARS-CoV-2 transmission risks for different surgical specialties, provides clarification on the appropriate use of PPE in the context of current specialty and national guidelines^13^, and discusses the ethical dilemma of PPE rationing that we are currently facing.

## Methods

This systematic review was undertaken in accordance with PRISMA guidelines^14^. MEDLINE (via PubMed), Embase and WHO COVID-19 databases were searched. Newspaper and internet article sources were identified using a media database called Nexis (https://www.lexisnexis.com/en-us/products/nexis.page). The search terms used were: (‘Surgeons’[mesh] AND (‘personal protective equipment’[mesh] AND ‘COVID-19’[mesh]). *Appendix S1* (supporting information) shows the search strategy for MEDLINE, which was adapted for other databases. There was no restriction on publication type. This search was complemented by an exhaustive review of the bibliography of key articles. Results were restricted to articles in the English language.

### Inclusion and exclusion criteria

All studies on the application of PPE in surgery during the COVID-19 pandemic were included. This included articles that reported on potential risks of transmission in surgery, types of PPE, specialty-specific risks and guidance. Articles that described PPE not in relation to surgery or COVID-19 were excluded, as were those not written in English.

### Data extraction and assessment of study quality

Two authors independently assessed the articles for inclusion and exclusion criteria and extracted data, with a third author resolving any differences. The data extraction was independently checked by the senior author. The following baseline data were extracted from each study: first author, year of publication, data collection period, geographical location and surgical specialty. Data were extracted on the sources of transmission in surgery, types of PPE recommended, surgical specialty-specific risks and considerations, PPE shortages and rationing.

### Narrative synthesis

Given the marked heterogeneity in study design and types that have been published during the emerging COVID-19 pandemic, a narrative synthesis was performed according to the guidance on the conduct of narrative synthesis in systematic reviews^15^. Three authors systematically summarized each article using bullet points to document key aspects of each study, focusing specifically on the availability, usage and rationing of PPE in surgery. The senior author identified and grouped common themes, divided larger themes into subthemes, tabulated a combined summary of the article, and synthesized a common rubric for each theme.

## Results

**Fig. 1:**
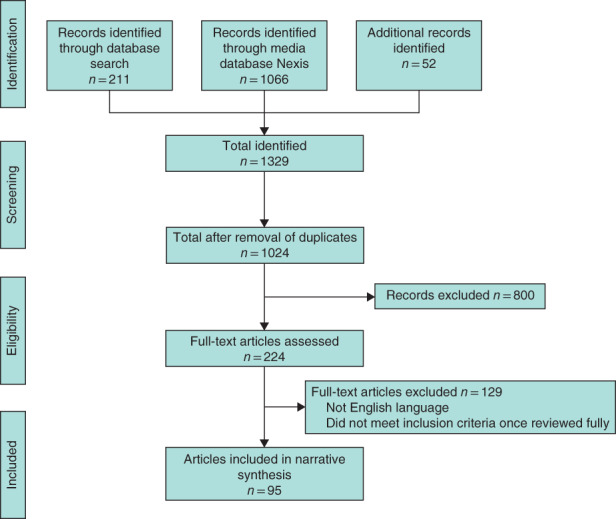
Flow diagram showing selection of articles for review

A total of 1329 articles were identified from all literature sources, leaving 1024 for screening of titles and abstracts following removal of duplicates. Ninety-five studies met the inclusion criteria and were included in the narrative synthesis (*Fig. 1* and *Table 1*).

**Table 1:** Studies identified for inclusion in narrative synthesis, grouped by theme

Reference	Year	Journal	Title
**Sources of transmission**			
Bahl *et al*.^16^	2020	*Journal of Infectious Diseases*	Airborne or droplet precautions for health workers treating coronovirus disease
Mowbray *et al*.^28^	2020	*BJS*	Safe management of surgical smoke in the age of COVID-19
Hensman *et al*.^29^	1998	*Surgical Endoscopy*	Chemical composition of smoke produced by high-frequency electrosurgery in a closed gaseous environment. An *in vitro* study
Karoo *et al*.^30^	2004	*Plastic and Reconstructive Surgery*	Surgical smoke without fire: the risks to the plastic surgeon
Neuman *et al*.^31^	2006	*Journal of Virology*	Supramolecular architecture of severe acute respiratory syndrome coronavirus revealed by electron cryomicroscopy
Johnson and Robinson^32^	1991	*Journal of Medical Virology*	Human immunodeficiency virus-1 (HIV-1) in the vapors of surgical power instruments
Sawchuk *et al*.^33^	1989	*Journal of the American Academy of Dermatology*	Infectious papillomavirus in the vapor of warts treated with carbon dioxide laser or electrocoagulation: detection and protection
Gloster and Roenigk^34^	1995	*Journal of the American Academy of Dermatology*	Risk of acquiring human papillomavirus from the plume produced by the carbon dioxide laser in the treatment of warts
Ling *et al*.^35^	2020	*Chinese Medical Journal*	Persistence and clearance of viral RNA in 2019 novel coronavirus disease rehabilitation patients
Mottrie^36^	2020	*European Association of Urology*	ERUS (EAU Robotic Urology Section) guidelines during COVID-19 emergency
Chen *et al*.^37^	2020	*Journal of Medical Virology*	The presence of SARS-CoV-2 RNA in feces of COVID-19 patients
Drosten *et al*.^38^	2003	*New England Journal of Medicine*	Identification of a novel coronavirus in patients with severe acute respiratory syndrome
Grant *et al*.^39^	2003	*New England Journal of Medicine*	Detection of SARS coronavirus in plasma by real-time RT–PCR
Ng *et al*.^40^	2003	*Clinical Chemistry*	Serial analysis of the plasma concentration of SARS coronavirus RNA in pediatric patients with severe acute respiratory syndrome
Ng *et al*.^41^	2003	*Clinical Chemistry*	Quantitative analysis and prognostic implication of SARS coronavirus RNA in the plasma and serum of patients with severe acute respiratory syndrome
Corman *et al*.^42^	2016	*Clinical Infectious Diseases*	Viral shedding and antibody response in 37 patients with Middle East respiratory syndrome coronavirus infection
Huang *et al*.^43^	2020	*Lancet*	Clinical features of patients infected with 2019 novel coronavirus in Wuhan, China
Chang *et al*.^44^	2020	*Transfusion Medicine Reviews*	Coronavirus disease 2019: coronaviruses and blood safety
Saadi *et al*.^20^	2020	*Otolaryngology – Head and Neck Surgery*	A commentary on safety precautions for otologic surgery during the COVID-19 pandemic
Zhu *et al*.^21^	2020	*Neurosurgery*	A COVID-19 patient who underwent endonasal endoscopic pituitary adenoma resection: a case report
Hsieh *et al*.^22^	2020	*Facial Plastic Surgery & Aesthetic Medicine*	A guide to facial trauma triage and precautions in the COVID-19 pandemic
Say *et al*.^23^	2020	*Journal of Pediatric Gastroenterology and Nutrition*	Risk stratification and PPE use in pediatric endoscopy during the COVID-19 outbreak: a single-center protocol
Verbeek *et al*.^24^	2020	*Cochrane Database Systematic Reviews*	Personal protective equipment for preventing highly infectious diseases due to exposure to contaminated body fluids in healthcare staff
Brown and Pope^19^	2020	*Anaesthesia*	PPE and possible routes of airborne spread during the COVID-19 pandemic
Takhar *et al*.^25^	2020	*European Archives of Oto-Rhino-Laryngology*	Recommendation of a practical guideline for safe tracheostomy during the COVID-19 pandemic
Wong *et al*.^17^	2020	*Journal of Hospital Infection*	Risk of nosocomial transmission of coronavirus disease 2019: an experience in a general ward setting in Hong Kong
Francis *et al*.^26^	2020	*Surgical Endoscopy*	SAGES and EAES recommendations for minimally invasive surgery during COVID-19 pandemic
Wang *et al*.^27^	2020	*International Journal of Infectious Diseases*	SARS-CoV-2 RNA detection of hospital isolation wards hygiene monitoring during the coronavirus disease 2019 outbreak in a Chinese hospital
Iacobucci^18^	2020	*British Medical Journal*	Covid-19: doctors performing resuscitation need higher level of PPE, says royal college
**Types of PPE**			
WHO^13^	2020		Rational use of personal protective equipment for coronavirus disease 2019 (COVID-19)
Public Health England^87^	2020		Consideration for acute personal protective equipment (PPE) shortages
WHO^46^	2020		Advice on the use of masks in the community, during home care, and in health care settings in the context of COVID-19: interim guidance 19 March 2020
Mizumoto *et al*.^47^	2020	*Eurosurveillance*	Estimating the asymptomatic proportion of coronavirus disease 2019 (COVID-19) cases on board the Diamond Princess cruise ship, Yokohama, Japan
Kim *et al*.^48^	2020	*Osong Public Health and Research Perspectives*	Identification of coronavirus isolated from a patient in Korea with COVID-19
Langrish *et al*.^49^	2009	*Particle and Fibre Toxicology*	Beneficial cardiovascular effects of reducing exposure to particulate air pollution with a simple face mask
Beckman *et al*.^50^	2013	*American Journal of Infection Control*	Evaluation of respiratory protection programs and practices in California hospitals during the 2009–2010 H1N1 influenza pandemic
Kelkar *et al*.^51^	2013	*International Journal of Infection Control*	How effective are face masks in operation theatre? A time frame analysis and recommendations
Zimmermann and Nkenke^64^	2020	*Journal of Cranio-Maxillofacial Surgery*	Approaches to the management of patients in oral and maxillofacial surgery during COVID-19 pandemic
**Specialty-specific considerations**			
Parikh *et al*.^73^	2020	*Journal of the American College of Surgeons*	Collaborative multi-disciplinary incident command at Seattle Children's Hospital for rapid preparatory pediatric surgery countermeasures to the COVID-19 pandemic
Leboulanger *et al*.^55^	2020	*European Annals of Otorhinolaryngology, Head and Neck Disease*	COVID-19 and ENT pediatric otolaryngology during the COVID-19 pandemic. Guidelines of the French Association of Pediatric Otorhinolaryngology (AFOP) and French Society of Otorhinolaryngology (SFORL)
Kowalski *et al*.^56^	2020	*Head and Neck*	COVID-19 pandemic: effects and evidence-based recommendations for otolaryngology and head and neck surgery practice
Workman *et al*.^52^	2020	*International Forum of Allergy & Rhinology*	Endonasal instrumentation and aerosolization risk in the era of COVID-19: simulation, literature review, and proposed mitigation strategies
Ciavattini *et al*.^71^	2020	*International Journal of Gynaecology and Obstetrics*	Expert consensus from the Italian Society for Colposcopy and Cervico-Vaginal Pathology (SICPCV) for colposcopy and outpatient surgery of the lower genital tract during the COVID-19 pandemic
Bann *et al*.^57^	2020	*Head and Neck*	Impact of coronavirus (COVID-19) on otolaryngologic surgery: a brief commentary
Carneiro *et al*.^74^	2020	*International Brazilian Journal of Urology*	Impact of the COVID-19 pandemic on the urologist's clinical practice in Brazil: a management guideline proposal for low- and middle-income countries during the crisis period
Syamal^60^	2020	*Laryngoscope Investigative Otolaryngology*	Literature-guided recommendations for otolaryngologists during the COVID-19 pandemic: a contemporary review
Kligerman *et al*.^53^	2020	*Head and Neck*	Managing head and neck cancer patients with tracheostomy or laryngectomy during the COVID-19 pandemic
British Dental Journal^62^	2020	*British Dental Journal*	OMFS and ENT surgeons issue new COVID-19 PPE guidance
Massey *et al*.^75^	2020	*Journal of the American Academy of Orthopaedic Surgeons*	Orthopaedic surgical selection and inpatient paradigms during the coronavirus COVID-19 pandemic
Walsh *et al*.^67^	2020	*Journal of Pediatric Gastroenterology and Nutrition*	Pediatric endoscopy in the era of coronavirus disease 2019: a North American Society for Pediatric Gastroenterology, Hepatology, and Nutrition position paper
Awad *et al*.^68^	2020	*Journal of the American Academy of Orthopaedic Surgeons*	Peri-operative considerations in urgent surgical care of suspected and confirmed COVID-19 orthopedic patients: operating rooms protocols and recommendations in the current COVID-19 pandemic
Fillingham *et al*.^76^	2020	*Journal of Arthroplasty*	Personal protective equipment: current best practices for orthopaedic teams
Frauenfelder *et al*.^58^	2020	*International Journal of Pediatric Otorhinolaryngology*	Practical insights for paediatric otolaryngology surgical cases and performing microlaryngobronchoscopy during the COVID-19 pandemic
Kimmig *et al*.^72^	2020	*Journal of Gynecologic Oncology*	Robot assisted surgery during the COVID-19 pandemic, especially for gynecological cancer: a statement of the Society of European Robotic Gynaecological Surgery (SERGS)
Crossley *et al*.^59^	2020	*Journal of Laparoendoscopic & Advanced Surgical Techniques*	Surgical considerations for an awake tracheotomy during the COVID-19 pandemic
Pawar *et al*.^69^	2020	*Journal of Laparoendoscopic & Advanced Surgical Techniques*	The technique and justification for minimally invasive surgery in COVID-19 pandemic: laparoscopic anterior resection for near obstructed rectal carcinoma
Day *et al*.^65^	2020	*Oral Oncology*	Head and neck oncology during the COVID-19 pandemic: reconsidering traditional treatment paradigms in light of new surgical and other multilevel risks
Diaz *et al*.^66^	2020	*American Journal of Surgery*	Elective surgery in the time of COVID-19
Impact News^70^	2020	*Impact News Service*	Updated general surgery guidance on COVID-19, 2nd revision, 7th April 2020
UK National Tracheostomy Safety Project^54^	2020		NTSP considerations for tracheostomy in the COVID-19 outbreak
Magennis and Kumar^61^	2020	*ENT UK*	Guidance PPE for patients with emergency oropharyngeal and nasopharyngeal conditions whose COVID status is unknown
Hettiaratchy^63^	2020	*British Association of Plastic, Reconstructive and Aesthetic Surgeons*	Highlights for surgeons from PHE COVID-19 IPC guidance
**PPE shortages and rationing**			
Rimmer^88^	2020	*British Medical Journal*	Covid-19: third of surgeons do not have adequate PPE, royal college warns
Patel *et al*.^77^	2020	*Head and Neck*	Early institutional head and neck oncologic and microvascular surgery practice patterns across the United States during the SARS-CoV-2 (COVID19) pandemic
Patel *et al*.^78^	2020	*Neurosurgery*	Letter: precautions for endoscopic transnasal skull base surgery during the COVID-19 pandemic
O'Sullivan^86^	2020	*British Medical Journal*	PPE guidance for covid-19: be honest about resource shortages
Ikonen^94^	2020	*Argus*	Surgeons told ‘not to risk health’ by working with inadequate PPE
Lockwood^95^	2020	*Northern Echo*	Coronavirus: surgeons told ‘not to risk health’ by working with inadequate PPE
Meechan^92^	2020	Chroniclelive.co.uk	Fewer than half of North East NHS surgeons and trainees say they have enough PPE; The Royal College of Surgeons surveyed 2000 surgeons and trainees around the UK to ask if they have enough protective equipment to fight covid-19
Hornall^81^	2020	*Press Association Mediapoint*	Dental surgeons voice concerns over shortage of PPE
Bowden and Connolly^93^	2020	*Press Association Mediapoint*	Surgeons told ‘not to risk health by working without PPE’
Clarke^84^	2020	*Breakingnews.ie*	Unsuitable PPE received from China will be replaced; surgeon warns medical staff making their own
Gammie^89^	2020	*Belfast Telegraph Online*	Third of surgeons do not have adequate protective equipment, survey finds
FARS New Agency^91^	2020	*FARS News Agency*	Survey finds one in three UK surgeons lacks enough protective kit
Berkovic^85^	2020	*The Australian*	Surgeons buy safety gear from Bunnings
Martinez^83^	2020	*NBC Chicago*	Construction company steps up after PPE stolen from Chicago surgeon's porch
Nevile^79^	2020	*Financial Times*	UK hospitals warn patients at risk due to shortage of gowns
Smyth *et al*.^80^	2020	*Times*	Coronavirus face masks for public ‘risk NHS shortage’
Roach^82^	2020	*London Evening Standard*	Group sourcing PPE for NHS trusts seeks help from fashion manufacturers amid shortage fears
Public Health England^87^	2020		Considerations for acute personal protective equipment (PPE) shortages
Campbell^90^	2020	*Guardian*	One in three UK surgeons lacks enough protective kit, survey finds
Ministero della Salute^96^	2020		Covid-19 – Situazione in Italia
Akst^97^	2020	*Scientist Magazine*	University of Washington pathology professor dies of COVID-19
Cockburn^98^	2020	*Independent*	Coronavirus: UK's first ‘frontline’ doctor dies after contracting disease
Ponsonby^99^	2020	*Health Service Journal*	No NHS staff member should die from work-acquired covid-19
**Ethics**			
Binkley and Kemp^106^	2020	*Journal of the American College of Surgeons*	Ethical rationing of personal protective equipment to minimize moral residue during the COVID-19 pandemic
Forrester *et al*.^101^	2020	*Journal of the American College of Surgeons*	Precautions for operating room team members during the COVID-19 pandemic
Gibbons^107^	2020	*Times*	Lifting lockdown will kill thousands, warn surgeons
Raphael^103^	2020	*Bloomberg*	Why surgeons don't want to operate right now
Wright^108^	2020	*Telegraph*	Calls for Government to investigate ‘alarming’ number of BAME deaths in health service
Brooks and Morris^109^	2020	*Guardian*	Scotland and Wales concerned over reports England is prioritised for coronavirus PPE
McGuinness^110^	2020	*Sky News*	Coronavirus: Minister ‘confident’ delayed shipment of protective equipment will arrive today
**Innovation**			
Turer *et al*.^115^	2020	*Journal of the American Medical Informatics Association*	Electronic personal protective equipment: a strategy to protect emergency department providers in the age of COVID-19
Erickson *et al*.^114^	2020	*Journal of Arthroplasty*	Helmet modification to PPE with 3D printing during the COVID-19 Pandemic at Duke University Medical Center: a novel technique
Dargaville *et al*.^112^	2020	*Polymer Degradation and Stability*	Opinion to address a potential personal protective equipment shortage in the global community during the COVID-19 outbreak

PPE, personal protective equipment.

### Sources of transmission during surgery

Transmission of SARS-CoV-2 takes place via particles or droplets containing the virus (larger than 5 μm, travel less than 1 m) as well as aerosol (smaller particles less than 5 μm, travel more than 1 m), via fomites and subsequent direct contact (touching eyes, nose or mouth)^16,17^. This is recognized to be especially high risk for healthcare professionals performing resuscitation^18^. PPE requirements have largely been defined based on whether a procedure is aerosol-generating or not^19^. Although airway procedures such as intubation, extubation and suctioning are widely recognized to generate aerosols, specific aerosol-generating procedures (AGPs) have been less well described, but are critical to identify during the COVID-19 pandemic to guide where extra PPE precaution is indicated (*Table 2*)^20–26^. A study^27^ showed that SARS-CoV-2 RNA from sewage samples was positive from inlets of the sewage disinfection pool and negative from the outlet of the last sewage disinfection pool, suggesting that strict disinfection and hand hygiene could decrease the hospital-associated COVID-19 infection risk to staff in isolation wards^27^.

**Table 2:** Summary of aerosol-generating procedures and guidelines for different surgical subspecialties

Specialty	Types of aerosol-generating procedure	Source
Cardiothoracic surgery	Sternotomy Thoracotomy Bronchoscopy	Society for Cardiothoracic Surgery https://scts.org/wp-content/uploads/2020/03/SCTS-ACTACC-SCPS-Theatre-COVID-pathway-Final.pdf Updated 23 March 2020
Ear, nose and throat	Tracheostomy (insertion/open suctioning/removal) Upper airway procedures involving suctioning Flexible nasal endoscopy and laryngoscopy Microsuction, management of epistaxis and tonsillitis/quinsy, airway emergencies Mastoid drilling	ENT UK https://www.entuk.org/guidance-ent-during-covid-19-pandemic Updated 16 March 2020 British Society of Otology https://www.entuk.org/guidance-undertaking-otological-procedures-during-covid-19-pandemic-0 Updated 25 March 2020
General surgery	Laparotomy – bowel content and diathermy Laparoscopy – smoke aerosols Endoscopy, especially upper gastrointestinal	Intercollegiate General Surgery Guidance on COVID-19 https://www.rcseng.ac.uk/coronavirus/joint-guidance-for-surgeons-v2/ Updated 7 April 2020
Maxillofacial surgery	High-speed drilling, e.g. facial trauma All intraoral procedures involving suctioning	British Association of Oral and Maxillofacial Surgeons https://www.baoms.org.uk/_userfiles/pages/files/professionals/covid_19/baoms_baos_covid_advice_update_25_march_2020_final.pdf Updated 25 March 2020
Neurosurgery	High-speed drilling/craniotomy Endonasal procedures	The Society of British Neurological Surgeons https://www.sbns.org.uk/index.php/policies-and-publications/covid/ Updated 2 April 2020
Paediatric surgery	As for general surgery, laparotomy and laparoscopy considered to be possible aerosol-generating procedures	British Association of Paediatric Surgeons – no specific PPE guidelines but directed to those of the Royal College of Surgeons of England https://www.rcseng.ac.uk/standards-and-research/standards-and-guidance/good-practice-guides/coronavirus/covid-19-good-practice-for-surgeons-and-surgical-teams/ Updated 3 April 2020
Plastic and reconstructive surgery	High-speed drilling/bone burring Dermatome for split-thickness skin graft Versajet in burns debridement	British Association of Plastic, Reconstructive and Aesthetic Surgeons http://www.bapras.org.uk/docs/default-source/covid-19-docs/ppe-guidance-for-plastic-surgeons---bapras-branding.pdf?sfvrsn=2
Trauma and orthopaedic surgery	High-speed drilling/sawing Bone debridement	British Orthopaedic Association http://www.boa.ac.uk/uploads/assests/ee39d8a8-9457-4533-9774e973c835246d/4e3170c2-d85f-4162-a32500f54b1e3b1f/COVID-19-BOASTs-Combined-FINAL.pdf Updated 21 April 2020
Urology	Laparoscopy and robot-assisted procedures Urethral or ureteral catheterization (recognized to cause coughing)	The British Association of Urological Surgeons https://www.baus.org.uk/about/coronavirus_covid-19.aspx Updated 25 March 2020
Vascular surgery	All arterial surgery Amputations	Vascular Society https://www.vascularsociety.org.uk/_userfiles/pages/files/Newsletters/2020/Presidents%20update%2027_03_20.pdf Updated 27 March 2020

PPE, personal protective equipment.

Other than AGPs, the risks of transmission include the fumes released during surgery and contaminated body fluids. Owing to the novelty of COVID-19 there is no definitive evidence to quantify the risks of transmission via smoke derived from open or laparoscopic surgery. However, there is enough evidence based on previous studies to support steps being undertaken to manage the potential risks^28^.

Electrocautery creates particles with the smallest mean size of 0·07 μm; laser tissue ablation generates larger particles with a mean size 0·31 μm, and the largest particles are generated by the ultrasonic (harmonic) scalpel, at 0·35–6·5 μm^29^. The smaller the particles, the further they travel. Smaller particles are more chemically based, but as the particulate matter increases in size, it poses more of a biological hazard, acting as a vector for pathogen transmission, with larger particles travelling up to 1 m from the operative field^30^. The majority of smoke evacuation and filtration modes facilitate capture of particles larger than 0·01 μm. As the SARS-CoV-2 aerodynamic size is described in the range of 0·06–0·15 μm^29,31^, the use of devices with smoke evacuation filters theoretically reduces local inoculation. Previous work has confirmed viral content (papillomavirus and human immunodeficiency virus) within surgical smoke^32,33^, and documented operator contraction of a papillomavirus using carbon dioxide laser^34^.

There are also risks of transmission owing to urine spillage and aerosolization in open and minimally invasive urological interventions, with Ling *et al*.^35^ reporting persistence of SARS-CoV-2 nucleic acid in urine. Although disease transmission of COVID-19 through urine has not yet been demonstrated, the European Association of Urology Robotic Urology Section^36^ stated that urethral or ureteral catheterization during laparoscopic and robotic procedures should be treated with caution. The British Association of Urological Surgeons has described the risk of operator contamination from urine splash as minimal and stated that aerosol risks are more significant from patients coughing during intimate procedures, such as catheterization, or following intubation/extubation. Furthermore, SARS-CoV-2 RNA has also been identified in faeces^37^.

Many studies have found that SARS-CoV-1 RNA can be detected in plasma, since the first report on 10 April 2003^38–40^. Overall, 78 per cent of patients had detectable viral RNA in the first week of their illness^41^, in line with data on MERS-CoV^42^ and SARS-CoV-2^43^. Owing to the risk of asymptomatic carriage and the presence of virus in the blood, the European Centre for Disease Prevention and Control (ECDC) and the American Association of Blood Banks have published rapid risk assessments regarding blood safety during the pandemic. The ECDC implied a precautionary deferral of donation of blood and cells for 21 days after possible exposure to a confirmed patient or by anyone who returned from Wuhan, China, applying the approach used for SARS-CoV-1 and MERS-CoV^44^.

### Types of personal protective equipment

**Fig. 2:**
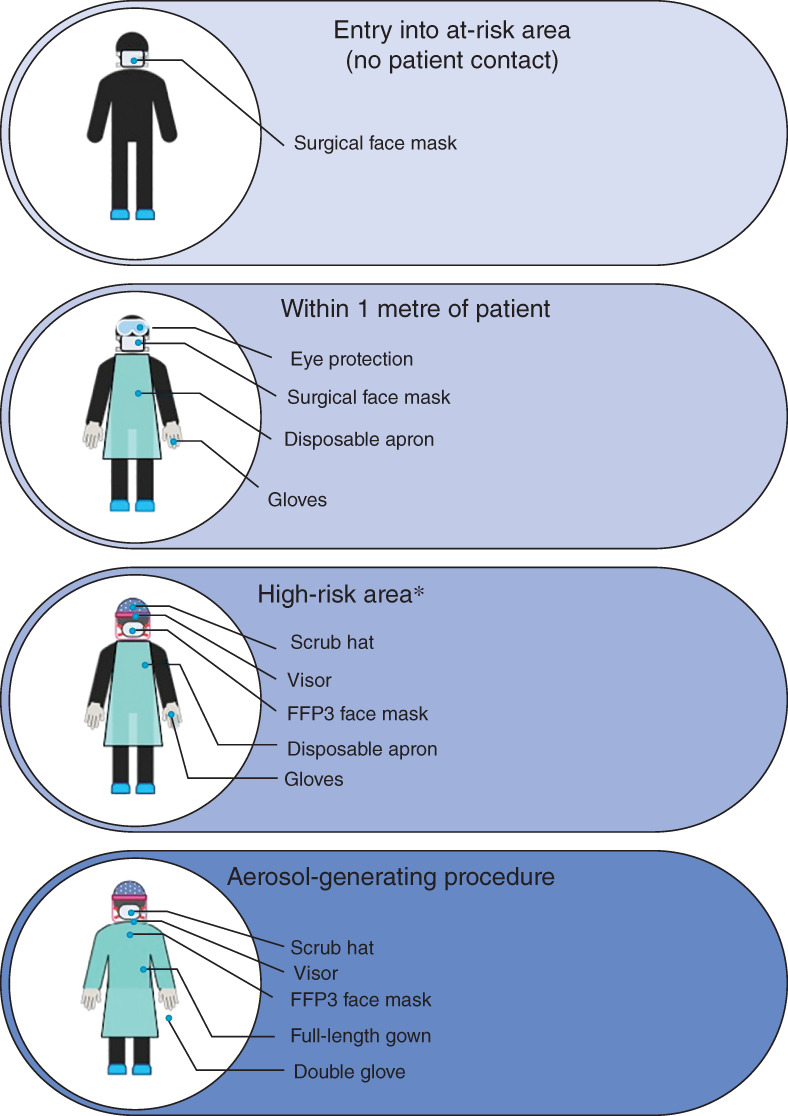
Personal protective equipment required in different surgical environments during COVID-19 pandemic

Recommendations made by the WHO regarding the use of PPE in the management of patients who have, or are suspected to have, COVID-19 in community and hospital settings have evolved alongside developing evidence regarding transmission^13^. The standard PPE guidance from Public Health England (PHE) for healthcare workers involved in the direct care (within 1 m) of patients with confirmed or suspected COVID-19 include: disposable apron, gloves, a fluid-repellent surgical mask and eye protection comprising either goggles or a face shield^45^. When working in high-risk units (ICU, high-dependency unit, accident and emergency, resuscitation, wards with non-invasive ventilation or continuous positive airway pressure ventilation, operating theatres, endoscopy units) or in the operating theatre where AGPs are being undertaken, a respirator (N99 or FFP3 equivalent, which can be either valved or unvalved) is recommended instead of a surgical mask, along with a fluid-repellent long gown^46^ and full-face shield or visor^13,45–46^ (*Fig. 2*). PHE is in support of full PPE in relation to AGPs conducted in *any* patient; given the high asymptomatic carrier rate of 16–50 per cent reported in some populations^47^, the assumption should be made that all patients, regardless of symptoms, are COVID-19-positive.

*ICU, high-dependency unit and areas where aerosol-generating procedures are carried out.

**Fig. 3:**
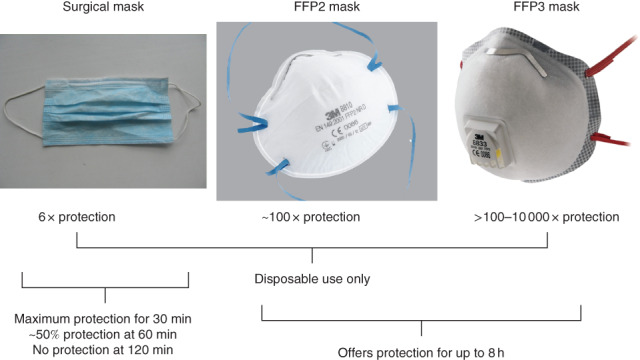
Types of face mask and their relative risk reduction

**Fig. 4:**
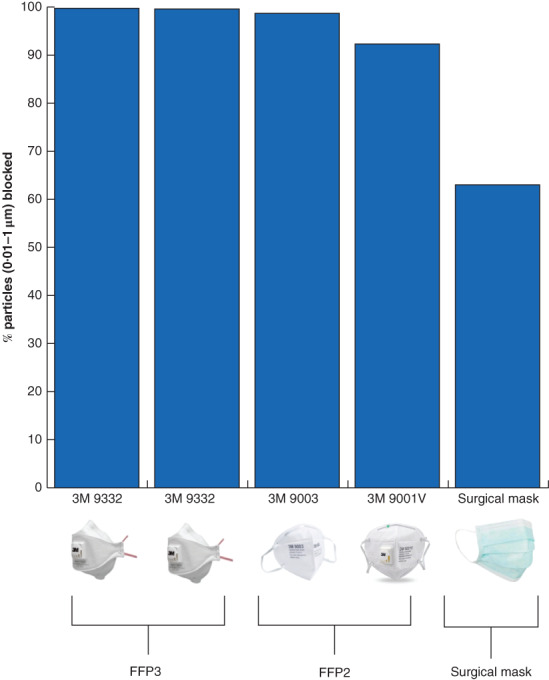
Percentage particles filtered (0·01–1 μm diameter) with different types of face mask

Respirator masks are categorized according to their ability to filter fine particles in the scale of 0·01–1 μm in size according to the European Union (EU)-defined Filtering Face Piece (FFP) scale, whereas SARS-CoV-2 measures 0·07–0·09 μm in diameter on electron microscopy^48^. FFP3 masks represent the standard of precaution, able to filter over 99 per cent (offering 100–10 000-fold protection), compared with 63 per cent (6-fold) for standard surgical masks^49^ (*Fig. 3*). Constraints within the National Health Service (NHS) have been recognized, and both FFP2 or N95-equivalent respirators offer high levels of protection if fitted well, with a minimum efficiency of 92–98 per cent (offering 100-fold protection) (*Fig. 4*). Protection using FFP2/3 respirators has been reported to last up to 8 h and current guidelines support sessional use in the care of multiple patients in red zones^50^, whereas protection has been reported to last 30 min for fluid-repellent surgical masks^51^. It is important to note that masks alone are not the panacea, and need to be combined with correct fitting via fit tests, avoidance of face/mask touching, regular hand washing and social distancing to minimize transmission^13^.

Data extracted from Kelkar *et al*.^51^ and https://www.hse.gov.uk/research/rrpdf/rr619.pdf.

Data extracted from Langrish *et al*.^49^ and graphs modified from https://smartairfilters.com/en/blog/comparison-mask-standards-rating-effectiveness/.

### Surgical specialty guidance for personal protective equipment

The current advice from UK surgical professional bodies is summarized in *Table 2*. Emerging evidence has highlighted that certain subspecialties are at higher risk based on their potential exposure to high viral loads in mucosal membranes of the nasopharynx and oropharynx, such as ear, nose and throat (ENT), maxillofacial, and plastic and reconstructive surgery of the head and neck^52,53^. Most specialty guidelines recommend avoidance of AGPs wherever possible, but full PPE is advised if this is required. The British Association for Paediatric Otolaryngology and ENT UK in conjunction with the National Tracheostomy Safety Project have outlined measures for tracheostomies in an effort to reduce potential risks. The documents highlight the requirement to balance the need for a tracheostomy *versus* the potential risks to both the patient and staff^54^. Many further publications relating to changes in ENT practice have been published^55–60^. The British Association of Oral and Maxillofacial Surgeons guidance recommends full PPE for all close face-to-face contact with patients, not only during treatment but also examination, until the current trajectory has flattened^61,62^. This can be applied to other surgical specialties such as plastic surgery and ENT, which involve examination and treatment of the head and neck area when managing trauma or cancer near the aerodigestive tract^63–65^.

In other specialties there has been a drive to identify high-risk procedures and minimize their use where possible^66^. In general surgery, laparoscopy and endoscopy should be performed only where there is no alternative and laparotomies carried out wearing full PPE^67–70^. Similar changes in guidance have been seen in gynaecology^71,72^, paediatric surgery^73^, urology^74^, orthopaedics^75,76^, head and neck cancer^77^ and neurosurgery^78^.

### Shortages of personal protective equipment

In the short history of this pandemic, medical resources have already been rapidly overwhelmed, including PPE. In recent days, there have been well reported critical shortages of gowns^79^ and masks^80–82^ in particular, with famous businesses supporting the national need in addition to other philanthropic donations^83,84^. In response to the shortage, there have been reports of healthcare workers resorting to procuring their own PPE^85^. The recognition, by the WHO and others, that the current global stockpile of PPE is inadequate to meet not only the current need but also future escalating demand^13^, has resulted in multiple changes to PPE guidelines published around the world, including PHE, which has been criticized for basing guidance on availability of resources rather than maximizing the safety of healthcare professionals^86^. The most recent changes, dated 17 April 2020, include PHE suggesting three options if the supply runs out, including sessional use of PPE, reusing it or using alternatives to standard PPE^87^. The shortage of PPE combined with unclear and changing guidance has resulted in anxiety and confusion for healthcare workers.

The Royal College of Surgeons of England (RCS) found that almost one-third (32·5 per cent) of UK surgeons do not have access to enough masks, gowns or eye protection, from a survey of 1978 surgeons and surgical trainees, and has described a widespread lack of PPE for frontline staff^88–92^. The RCS recognizes that surgeons should not risk their health if they have inadequate PPE^93–95^. In response to the PPE shortage, ENT UK^61^ highlighted that although ‘FFP3 masks are a precious life-saving and protecting resource, clinical staff are also life-saving resources and deserve the best protection we can offer’.

The medical workforce is at high risk of exposure as well as increased viral infective dose, thought to translate to viral load, an independent factor in the severity of the illness. In Italy, healthcare workers including several surgeons experienced high rates of infection and death owing an early lack of PPE^96^. There have also been early deaths, again including surgeons, among healthcare workers reported in the USA and the UK^97,98^. There is good evidence that improved access and use of PPE vastly reduced healthcare worker infections in both Italy and China^99^.

### Ethical dilemma of personal protective equipment rationing

Healthcare resource allocation has political, economic and moral dimensions, and rationing is inevitable in a system where there are limited resources. The National Institute for Health and Care Excellence routinely rations treatments based on quality-adjusted life-years (QALYs), which represent beneficial health activity; the lower the cost per QALY, the more efficient the healthcare activity^100^. Rationing of PPE adds a new dimension to modern healthcare practice. We are no longer rationing between patients but between healthcare workers, with organizations having to respond to the changing situation on the ground^66,77–78,101^. Ethical guidance regarding justice within healthcare rationing has often been written by health economists^102^; professional philosophers have been reluctant to tackle day-to-day policy questions, reflecting the great difficulty in forming ethically sound answers^102^.

There has always been an acceptance that working in a healthcare setting carries a level of personal risk; however, it would seem unreasonable for a healthcare worker to carry out a healthcare activity if there were a high risk of death^103^. In the Ebola crisis, 58·3 per cent of healthcare workers infected with Ebola died in the three worst affected countries^104^. In reference to the General Medical Council's best practice of ‘making the care of your patient your first concern’, in his book *Tough Choices*, Sokol^105^ stated that in ‘extreme circumstances – such as epidemics, where treating patients involves a high risk of infection and modest benefits to patients – doctors’ obligations to their children, parents, siblings and loved ones take priority over the care of patients'.

A doctor's duty of care creates an implicit and explicit social contract with both patients and society as a whole, to care for and treat patients despite a degree of personal risk^102^. PHE and other public health bodies have suggested a minimum level of PPE for specific interactions with patients with, or suspected to have, COVID-19 (*Fig. 2*) and, combined with variations in guidance from different national and international bodies with differing local interpretations of guidance and poor communication, a sense of fear has understandably been created among healthcare professionals.

Guidelines for the rationing of PPE should follow the scientific evidence and also be morally defensible. A utilitarian approach, where the greatest good for the greatest number is promoted, provides a valuable ethical framework and is intuitively scientific as it focuses on the consequences, as being the morally significant entity. It is scientifically justifiable to provide maximal PPE for higher-risk procedures such as AGPs. Some clinicians are better placed to treat patients with COVID-19 than others. A utilitarian approach would support protecting these people who could provide the most benefit. Other clinicians may be offered lower levels of protection or their work activities reduced to limit the demand on PPE supplies. To limit certain activities is acceptable; however, to discriminate between clinicians would be a difficult moral calculation and likely to cause considerable distress^106^.

Other ethical considerations such as reciprocity and social worth have also been proposed^106^. Reciprocity involves giving more protection to clinicians who are most vulnerable owing to the higher level of risk that their job entails. For example, intensivists and anaesthetists who regularly perform aerosolizing procedures may deserve the maximum level of protection, not just from a scientific perspective but also a moral one. Social worth is not typically seen as a morally permissible way of allocating resources. In extreme situations, such as a pandemic, where certain roles are essential for the functioning of society, it may become permissible. The idea of key workers being allowed to work and receive special benefits has been derived from this concept.

Protecting the vulnerable is another important ethical consideration. People with significant underlying health conditions, such as cancer or immune deficiencies, have been advised to shield and stay home, and lifting lockdown too early will result in an excessive number of deaths^107^. However, with emerging evidence that black, Asian and minority ethnic (BAME) groups are at higher risk of severe COVID-19 infection, it is imperative that this is urgently investigated, and guidance is developed to protect healthcare workers in at-risk groups^108^.

A utilitarian perspective, using scientific evidence and principles such as social worth, reciprocity and protection of the vulnerable, provides a useful framework for making difficult rationing decisions regarding PPE^106^. No one system of allocation will be acceptable to all^109^. Recent reports of reusing/washing gowns in circumstances of acute shortages of PPE and delays in international shipments^96,110^ have brought into sharp focus the difficult decisions that lie ahead. It is imperative that decisions and the rationale behind them are transparent and collaborative with all relevant national bodies. This will limit the amount of moral residue and help with rebuilding after the pandemic is over^105^.

### Innovation

Owing to global issues with the supply chain of PPE, many countries have looked to innovative solutions. An initial focus has been on producing reusable PPE, to reduce both the economic and environmental impact (28 per cent reduction in natural resource energy consumption and 93 per cent reduction in solid waste generation)^110,111^, alongside the exploration of simple measures that could aid personal ownership of items and tracking of individual use in hospital settings, increasing the acceptance of resterilized items^112^.

The European Association for Additive Manufacturing has responded to a request from the European Commission to help produce medical equipment for hospitals tackling the COVID-19 outbreak^113^ and, in the UK, emergency working groups have been set up: the Sustainable Hub for Innovation, Execution, Launch and Distribution in England, and the South Wales Additive and Rapid Manufacturing Consortium in Wales. These groups have brought industry leaders, scientists and government together in an umbrella collaboration to create innovative solutions to meet the PPE demand, including cutting edge printing hubs. In addition to producing new equipment, novel repurposing of existing theatre equipment has been undertaken, such as adaptation of orthopaedic helmet systems used for elective arthroplasties, where manifolds were 3D-printed and hoods sewn on to the helmet^114^.

There have even been attempts to create electronic PPE by using telemedicine tools to perform electronic medical screening examinations, which has the potential to conserve PPE and protect providers while maintaining safe standards for medical screening^115^.

## Discussion

In the short history of this pandemic, medical resources have been rapidly overwhelmed. There has been a huge focus both in the NHS itself and the national press regarding PPE. Lessons from China showed that high healthcare worker infection rates were only improved once PPE was adequate. As a nation, we have had time to plan for this pandemic compared with other less fortunate countries such as Italy and China, where relatively large numbers of healthcare workers succumbed to COVID-19. Such planning has been widely criticized in the scientific press^116^, and in recent days there have been well reported critical shortages of PPE in the UK including gowns and masks. After missing out on an opportunity to join the EU joint procurement scheme to bulk-purchase PPE, there has been an ever more urgent need to secure and coordinate supply chains^117^. Boris Johnson has recently appointed a PPE ‘tsar’, former London Olympics chief executive Paul Deighton, to coordinate, source and boost manufacturing of protective equipment needed to protect NHS staff.

The medical workforce is at high risk of exposure as well as increased viral load and, although there is a need to balance limited supplies with staff and patient safety, this should not leave the healthcare professionals treating patients with inadequate PPE. The British Medical Association, RCS and Royal College of Nursing have all spoken out, saying that their members should not be in this position. We are learning more about the novel pathogen SARS-CoV-2 as worldwide research is carried out and shared, including identifying the staff most at risk – men, those with high BMI, increased age and co-morbidities, and those from the BAME community^118^. National guidelines must keep pace as new information comes to light to protect those most at risk.

These are unprecedented times when difficult decisions need to be made. However, in the case of rationing PPE, these decisions should be transparent, collaborative, accountable and adaptable as evidence of the pandemic evolves, rather than disguised as guidelines with the minimum level of protection. Changes in guidelines should be communicated honestly and clearly to the public and frontline healthcare professionals without political spin or ambiguity. Making morally sound policies is equally as important as following the scientific evidence to maintain trust, solidarity and a functioning society. Until there is a vaccine or proven treatments available, the requirement for surgeons to limit their workload and take sensible precautions is imperative in reducing transmission, flattening the curve, protecting themselves and patients, and reducing the death toll.

## Supplementary Material

BJS_11750_SI_Appendix_1 Appendix 1 Medline
