# Supplementary material for: Personal protective equipment for surgeons during COVID-19 pandemic: systematic review of availability, usage and rationing
Source: Br J Surg. 2020 Aug 24;107(10):1262–80. doi: 10.1002/bjs.11750 (PMC7273092; doi:10.1002/bjs.11750)
Supplement: BJS_11750_SI_Appendix_1 — Appendix 1 Medline [file bjs_11750_si_appendix_1.docx]

**Appendix 1**

**Medline**

| 1 | exp *betacoronavirus/ or exp *Coronavirus infection/ | 12397 |
| --- | --- | --- |
| 2 | ((corona* or corono*) adj1 (virus* or viral* or virinae*)).ti,ab. | 519 |
| 3 | ((novel or new or nouveau or "2019") adj2 (coronavirus* or "corona virus*" or coronovirus* or coronavirinae*)).ti,ab. | 3059 |
| 4 | (Wuhan* or Hubei* or Huanan or "2019-nCoV" or 2019nCoV or nCoV2019 or "nCoV-2019" or "COVID-19" or COVID19 or "CORVID-19" or CORVID19 or "WN-CoV" or WNCoV or "HCoV-19" or HCoV19 or CoV or "2019 novel*" or Ncov or "n-cov" or "SARS-CoV-2" or "SARSCoV-2" or "SARSCoV2" or "SARS-CoV2" or SARSCov19 or "SARS-Cov19" or "SARSCov-19" or "SARS-Cov-19" or Ncovor or Ncorona* or Ncorono* or NcovWuhan* or NcovHubei* or NcovChina* or NcovChinese*).ti,ab. | 15592 |
| 5 | (("seafood market*" or "food market*") adj10 (Wuhan* or Hubei* or China* or Chinese* or Huanan*)).ti,ab. | 58 |
| 6 | ((outbreak* or wildlife* or pandemic* or epidemic*) adj1 (China* or Chinese* or Huanan*)).ti,ab. | 82 |
| 7 | 1 or 2 or 3 or 4 or 5 or 6 | 24502 |
| 8 | exp Personal Protective Equipment/ | 29240 |
| 9 | personal protective equipment.ti,ab. | 2620 |
| 10 | PPE.ti,ab. | 3220 |
| 11 | 8 or 9 or 10 | 33440 |
| 12 | exp Surgeons/ | 8121 |
| 13 | exp Operating Room Nursing/ or exp Operating Room Technicians/ or exp Operating Rooms/ | 19271 |
| 14 | exp Surgical Procedures, Operative/ | 3112240 |
| 15 | Surgeon*.ti,ab. | 91384 |
| 16 | surgery.ti,ab. | 1113665 |
| 17 | surgical.ti,ab. | 947809 |
| 18 | 12 or 13 or 14 or 15 or 16 or 17 | 3986558 |
| 19 | 7 and 11 and 18 | 52 |
